# Reevaluating the two-representation model of numerical magnitude processing

**DOI:** 10.3758/s13421-015-0542-2

**Published:** 2015-08-13

**Authors:** Ting Jiang, Wenfeng Zhang, Wen Wen, Haiting Zhu, Han Du, Xiangru Zhu, Xuefei Gao, Hongchuan Zhang, Qi Dong, Chuansheng Chen

**Affiliations:** School of Psychology, Beijing Normal University, Beijing, China; Beijing Key Laboratory of Applied Experimental Psychology, Beijing Normal University, Beijing, China; Medical Supplies Depot, Academy of Military Medical Sciences, Beijing, China; Department of Psychology, University of Notre Dame, Notre Dame, IN USA; Department of Psychology, Henan University, Kaifeng, China; Max Planck Institute for Psycholinguistics, Nijmegen, The Netherlands; School of Social Development, Central University of Finance and Economy, Beijing, China; State Key Lab of Cognitive Neuroscience and Learning, Beijing Normal University, Beijing, China; Department of Psychology and Social Behavior, University of California, Irvine, CA USA

**Keywords:** Arabic numeral, Distance effect, Congruity effect, Number representation, Stroop interference

## Abstract

One debate in mathematical cognition centers on the single-representation model versus the two-representation model. Using an improved number Stroop paradigm (i.e., systematically manipulating physical size distance), in the present study we tested the predictions of the two models for number magnitude processing. The results supported the single-representation model and, more importantly, explained how a design problem (failure to manipulate physical size distance) and an analytical problem (failure to consider the interaction between congruity and task-irrelevant numerical distance) might have contributed to the evidence used to support the two-representation model. This study, therefore, can help settle the debate between the single-representation and two-representation models.

Many studies have documented the distance effect in numerical processing (Banks & Flora, 1977; Moyer & Landauer, 1967; Schwarz & Heinze, 1998): Number pairs that are farther apart in magnitude (e.g., 2 vs. 8) are easier to discriminate than number pairs that are closer to each other in magnitude (e.g., 2 vs. 3). The distance effect occurs even when participants are not required to process the magnitude information (e.g., Besner & Coltheart, 1979; Dehaene & Akhavein, 1995; Duncan & McFarland, 1980). For example, Dehaene and Akhavein asked participants to decide whether two numbers were physically the same (e.g., 2 2 or TWO TWO) or different (2 3 or 2 TWO). Although magnitude information was not needed to perform this same–different task, participants still showed the distance effect, suggesting that magnitude information was automatically processed. On the basis of such evidence, Dehaene and Akhavein (1995) proposed that numbers’ magnitude information has a single mental representation (i.e., one part of their triple-code model) that is automatically processed, even if it is not required in a given task.

However, not all studies have shown automatic processing of numbers’ magnitude information when it is task-irrelevant (e.g., Ganor-Stern & Tzelgov, 2008; García-Orza, Perea, Abu Mallouh, & Carreiras, 2012; Goldfarb, Henik, Rubinsten, Bloch-David & Gertner, 2011; Rubinsten, Henik, Berger, & Shahar-Shalev, 2002). For example, using the selection version (i.e., comparing two numbers presented on the screen) of the number Stroop task, Rubinsten et al. did not find the numerical distance effect for physical size judgments (i.e., which number is bigger in physical size?), although they did find the numerical distance effect for numerical magnitude judgments (i.e., which of the two presented numbers is bigger in numerical magnitude?). These results are inconsistent with Dehaene and Akhavein’s (1995) model of single representations of number magnitude. Therefore, Rubinsten et al. proposed a two-representation model of numerical magnitude processing. The first representation is an internal number line, which is subject to the distance effect. When making numerical comparisons, the participants apply algorithm-based processes to retrieve the required information from the mental number line. The second representation is composed of specific instances of Arabic numeral pairs. According to Rubinsten et al., after years of schooling and using numbers, adults have accumulated many instances of comparisons between number pairs, and can thus retrieve such number pairs directly. The processing of directly retrieved number pairs would not be subject to the distance effect.

However, we suspected that two aspects of Rubinsten et al.’s (2002) study might have contributed to their results. First, they did not examine potential interactions between congruity and numerical distance. Such interactions might have led to a nonsignificant main effect of task-irrelevant numerical distance because the positive distance effect under the congruent condition would cancel out the negative distance effect under the incongruent condition. Indeed, in their study using the classification version (i.e., comparing a number presented on the screen to a standard reference—e.g., 5) of the number Stroop paradigm, Schwarz and Ischebeck (2003) found two significant interactions. One was the overadditive interaction between congruity and task-relevant distance: Increasing the distance of the relevant attribute from close to far decreased the congruity effect (see their Fig. 4, p. 513). The other was the underadditive interaction between congruity and task-irrelevant distance: Increasing the distance of the irrelevant attribute from close to far enhanced the congruity effect (see their Fig. 5, p. 513). In the present study, we extended Schwarz and Ischebeck’s procedure of data analysis to the selection version of the Stroop paradigm (used by Rubinsten et al., 2002), to investigate whether potential interactions might have confounded Rubinsten et al.’s results.

Second, like most previous studies using the number Stroop paradigm, Rubinsten et al.’s (2002) experimental design was asymmetrical. That is, the reaction times (RTs) in the physical size judgment task were much shorter than those in the numerical magnitude judgment task, with an RT difference of about 100 ms. Other studies have found even bigger differences, such as 125 ms in Henik and Tzelgov (1982) and 180 ms in Girelli, Lucangeli, and Butterworth (2000). In other words, this asymmetry stacked the deck against the slower numerical-value dimension interfering with the faster physical-size dimension (MacLeod, 1991). In addition, most of these number Stroop studies have had more levels of numerical distance than of physical size distance (or physical size ratio). For example, the numerical distance has been given two (e.g., Besner & Coltheart, 1979; Tzelgov, Meyer, & Henik, 1992; Vaid, 1985; Vaid & Corina, 1989), three (e.g., Girelli et al., 2000; Henik & Tzelgov, 1982), or more than three (e.g., Zhou et al., 2007) levels, but physical size distance has had just one level. More recently, Cohen Kadosh, Henik, and Rubinsten (2008) controlled for this asymmetry in the design of their Experiment 2, by creating three numerical distances and three physical distances, which matched the RTs for the numerical and physical comparisons and achieved a symmetrical design (see also Algom, Dekel, & Pansky, 1996; Leibovich, Diesendruck, Rubinsten, & Henik, 2013; Pansky & Algom, 1999, 2002). Cohen Kadosh et al. did not find a main effect of numerical distance in physical comparisons, but they did find an interaction between congruity and task-irrelevant numerical distance. Unfortunately, they did not report separately the negative and positive effects of numerical distance for the congruent and incongruent conditions, and did not examine whether such effects were modulated by the physical size distance.

To overcome the two limitations mentioned above, in the present study we manipulated physical size distance systematically (with six levels; see the Method section for details). This design would allow us to determine whether the absence or the small effects of numerical distance in physical comparisons reported in previous studies were due to a lack of manipulation of physical distance. When more levels of physical size distance were used, we expected that both positive and negative effects of numerical distance would become more salient, because they would systematically affect the speed of processing of both numerical value and physical size (Algom et al., 1996; Noël, Rousselle, & Mussolin, 2005). In the case of the numerical task, both single- and two-representation models predicted a significant numerical distance effect, because the numerical value was task-relevant.

## Method

### Participants

Thirty-six right-handed undergraduate volunteers (22 female and 14 male; mean age = 22.4 years, ranging from 20 to 25 years) from Beijing Normal University were recruited for this study. All participants had normal eyesight in both eyes and gave written informed consent before the experiment.

### Stimuli and tasks

**Fig. 1:**
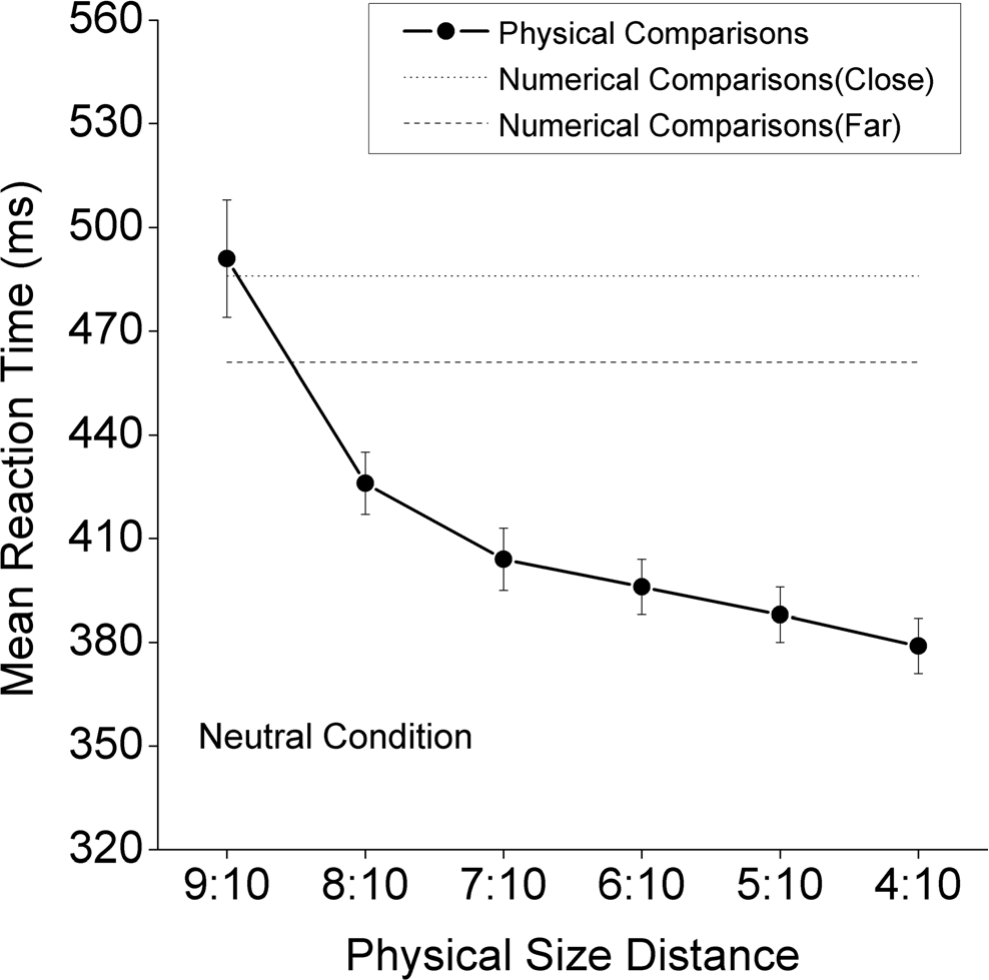
Mean reaction times for the neutral condition in the numerical task (numerical distance: close vs. far), and mean reaction times and standard errors for neutral conditions in the physical task (six physical size distances represented by six ratios)

All participants performed both the numerical magnitude and physical size judgment tasks. In the numerical magnitude judgment task (referred to as the *numerical task* hereafter), they were asked to judge the numerical value (magnitude) of Arabic numbers, and in the physical size judgment task (referred to as the *physical task* hereafter), they were asked to judge the physical size of Arabic numbers. For each task, 432 pairs of digits were used as the stimuli (144 each for the congruent, neutral, and incongruent conditions). A *congruent* stimulus was defined as a pair of digits in which a given digit was larger in both the numerical and physical dimensions (e.g., 3 ). An *incongruent* stimulus was defined as a pair of digits in which a given digit was larger in one dimension and smaller in the other dimension (e.g.,  7). The *neutral* stimuli were different in the two comparison tasks. For the physical task, the neutral stimuli included the same digit presented in two different physical sizes (e.g., 3 ). For the numerical task, the neutral stimuli included different numbers of the same physical size (e.g., 3 7). There were two numerical distance levels: close (2–3, 3–4, 2–4, 7–8, 8–9, and 7–9) and far (2–7, 2–8, 3–7, 3–9, 4–8, and 4–9). Likewise, there were six different physical size distance levels: 9:10, 8:10, 7:10, 6:10, 5:10, and 4:10, in terms of the heights of the digits. The biggest stimulus size for the numbers was 2.29° (vertical) × 1.43° (horizontal)—about 16 mm in height and 10 mm in width in Arial font. The smallest stimulus size for the numbers was 0.86° (vertical) × 0.57° (horizontal)—about 6 mm in height and 4 mm in width. Selection of the physical size distances (the number and the range) was based on a pilot study conducted to ensure that the six levels would cover all relative speed difference patterns illustrated in MacLeod’s review (1991; see his Fig. 1, p. 189).

Participants were seated about 40 cm from the computer screen. To counterbalance, one half of the participants (randomly selected) performed the numerical task first, followed by the physical task, and the other half completed the tasks in the reverse order. Before each task began, participants were given a block of 12 practice trials, and they were permitted to start the task only if they showed a thorough understanding of the task instructions (with more than 90 % accuracy for the practice trials). The instructions emphasized both speed and accuracy. Each trial began with the presentation of a fixation point “O” for 500 ms. After the disappearance of the fixation point, a pair of digits (15 mm apart from each other) appeared and remained on the screen until the participant pressed a key. The digits were white characters on a black background. Participants were asked to press the LEFT key if the left numeral was bigger, and to press the RIGHT key if the right numeral was bigger. A new trial began 1,000 ms after the participant responded.

### Statistical analysis

Repeated measures analyses of variance (ANOVAs) were conducted using Bonferroni correction, and the ensemble-adjusted *p* values are reported. The independent variables were task (numerical vs. physical comparison), congruity (congruent vs. incongruent), numerical magnitude distance (close vs. far), and physical size distance (9:10, 8:10, 7:10, 6:10, 5:10, and 4:10). RT outliers (beyond 3 *SD*s; about 1.47 % of trials for the numerical task, and about 2.12 % for the physical task) were excluded from the analyses. Neutral trials were excluded from ANOVAs because they had zero physical or numerical distance. After the four-way ANOVA showed significant omnibus effects, we followed up with a three-way ANOVA for each task to help us interpret the results. To ensure that the omnibus Type I error rate was .05, we used Bonferroni correction to adjust the *p* values.

## Results

**Fig. 2:**
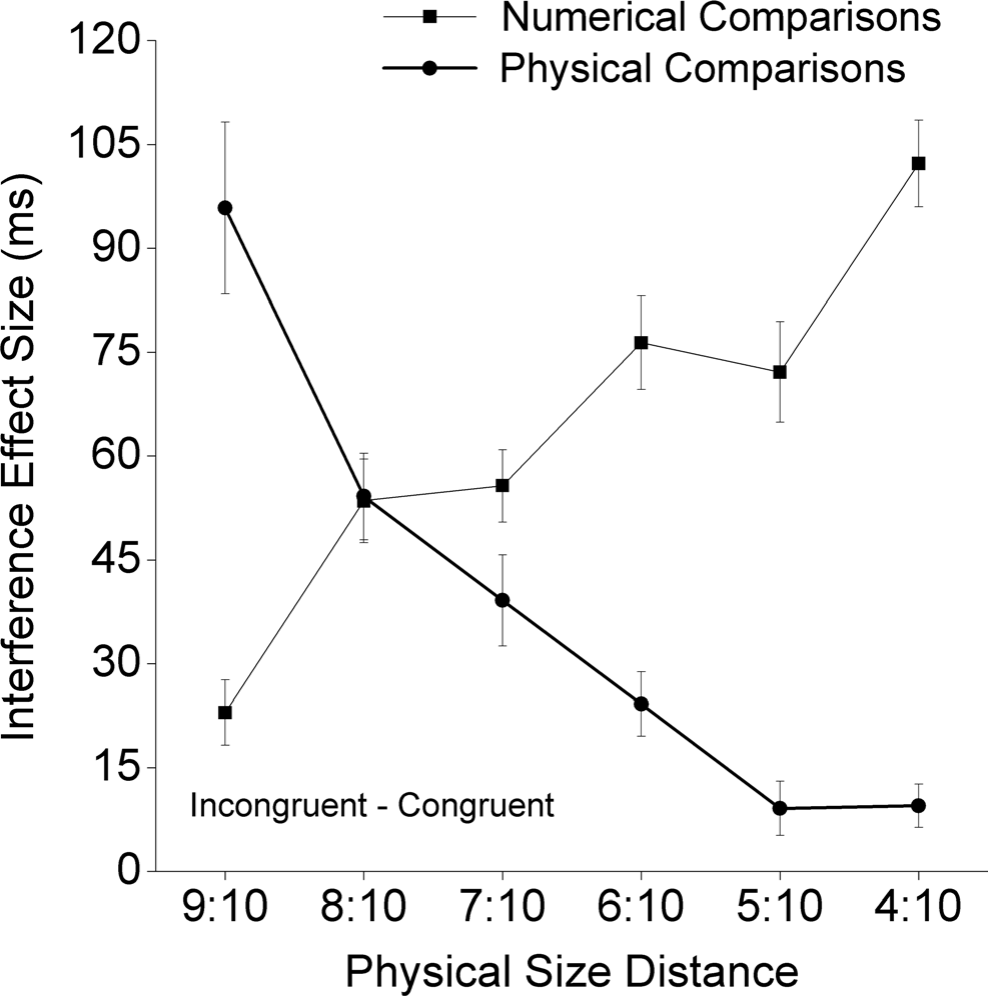
Congruity effect sizes (RT_incon_ – RT_con_) and standard errors in both the numerical and physical tasks as a function of physical size distances

The mean RTs for the numerical task are shown in Table 1, and those for the physical task are shown in Table 2. The mean RTs for the neutral conditions in both the numerical and physical tasks are shown in Fig. 1. The effect sizes of congruity as a function of physical size distance are shown in Fig. 2. Then, a 2 (task) × 2 (congruity) × 2 (numerical distance) × 6 (physical size distance) ANOVA was conducted on the RT data, with the neutral conditions excluded. Numerical comparison was significantly slower than physical comparison (483 vs. 426 ms) [*F*(1, 35) = 30.73, *p* < .001]. We also observed a main effect of congruity [*F*(1, 35) = 291.49, *p* < .001]. The interaction between congruity and task was significant [*F*(1, 35) = 14.89, *p* < .001], with significant congruity effects being detected in both tasks [numerical task, *F*(1, 35) = 190.30, *p* < .001; physical task, *F*(1, 35) = 66.61, *p* < .001]. The main effect of numerical distance was significant [*F*(1, 35) = 56.70, *p* < .001], and the simple effects of numerical distance were significant for both tasks [numerical task, *F*(1, 35) = 111.92, *p* < .001; physical task, *F*(1, 35) = 5.50, *p* < .024], even though the interaction between numerical distance and task was also significant [*F*(1, 35) = 82.21, *p* < .001]. Similarly, the main effect of physical size distance was significant [*F*(5, 175) = 57.96, *p* < .001], as were the simple effects of physical distance for both tasks [numerical task, *F*(5, 31) = 18.63, *p* < .001; physical task, *F*(5, 31) = 31.40, *p* < .001], even though the interaction between physical size distance and task was also significant [*F*(5, 175) = 96.00, *p* < .001]. Finally, although this was tangential to our hypotheses, all other interactions except for two were significant. Specifically, the significant interactions were the ones between congruity and physical size distance [*F*(5, 175) = 4.00, *p* < .002]; between numerical distance and physical size distance [*F*(5, 175) = 3.10, *p* < .05]; among task, congruity, and numerical distance [*F*(1, 35) = 91.54, *p* < .001]; among task, congruity, and physical size distance [*F*(5, 175) = 66.60, *p* < .001]; among congruity, numerical distance, and physical size distance [*F*(5, 175) = 7.28, *p* < .001]; and among task, congruity, numerical distance, and physical size distance [*F*(5, 175) = 10.80, *p* < .001]. The two nonsignificant interactions were the interaction between congruity and numerical distance, and the one among task, numerical distance, and physical size distance (both *p*s > .30).

**Table 1:** Mean reaction times and standard errors for each condition in the numerical task

	Congruent		Neutral		Incongruent	
	Close	Far	Close	Far	Close	Far
			Numerical Task			
9:10	464 ± 11	452 ± 12			505 ± 11	457 ± 10
8:10	466 ± 9	439 ± 9			521 ± 12	491 ± 11
7:10	465 ± 13	444 ± 10			540 ± 12	485 ± 11
6:10	452 ± 11	431 ± 11			552 ± 11	489 ± 11
5:10	465 ± 12	440 ± 11			539 ± 11	511 ± 11
4:10	459 ± 11	429 ± 10			562 ± 12	533 ± 10
1:1			486 ± 10	461 ± 10		

Physical size distances are represented by ratios

**Table 2:** Mean reaction times and standard errors for each condition in the physical task

	Congruent		Neutral	Incongruent	
	Close	Far		Close	Far
			Physical Task		
9:10	488 ± 21	458 ± 19	491 ± 17	545 ± 19	590 ± 25
8:10	419 ± 11	413 ± 11	426 ± 9	454 ± 14	486 ± 15
7:10	389 ± 9	394 ± 8	404 ± 9	423 ± 12	435 ± 12
6:10	393 ± 8	383 ± 7	396 ± 8	410 ± 11	411 ± 10
5:10	391 ± 8	386 ± 9	388 ± 8	389 ± 9	404 ± 12
4:10	387 ± 8	389 ± 8	379 ± 8	394 ± 9	400 ± 11

Physical size distances are represented by ratios

After finding the significant main and interactive effects, we proceeded to test directly whether the task-irrelevant distance effect occurred under either the congruent or incongruent conditions or under both, and examined whether these effects depended on task relevance. Specifically, Congruity (congruent vs. incongruent), Numerical Distance (close vs. far), and Physical Size Distance (9:10, 8:10, 7:10, 6:10, 5:10, and 4:10) were entered as main within-subjects factors in ANOVAs for the numerical and physical tasks separately.

**Fig. 3:**
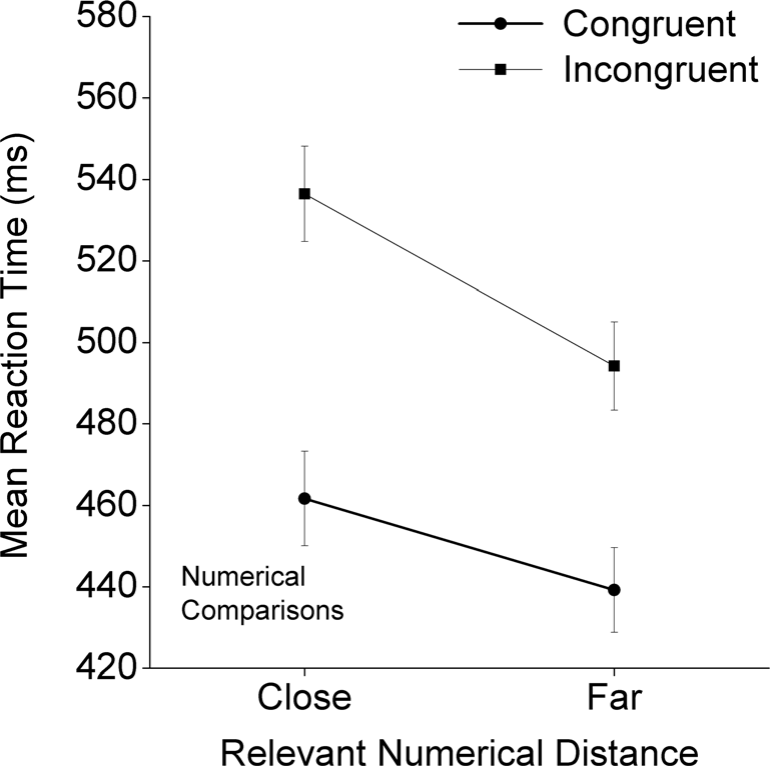
Mean reaction times and standard errors in the numerical task as a function of congruity and the task-relevant numerical distance

**Fig. 4:**
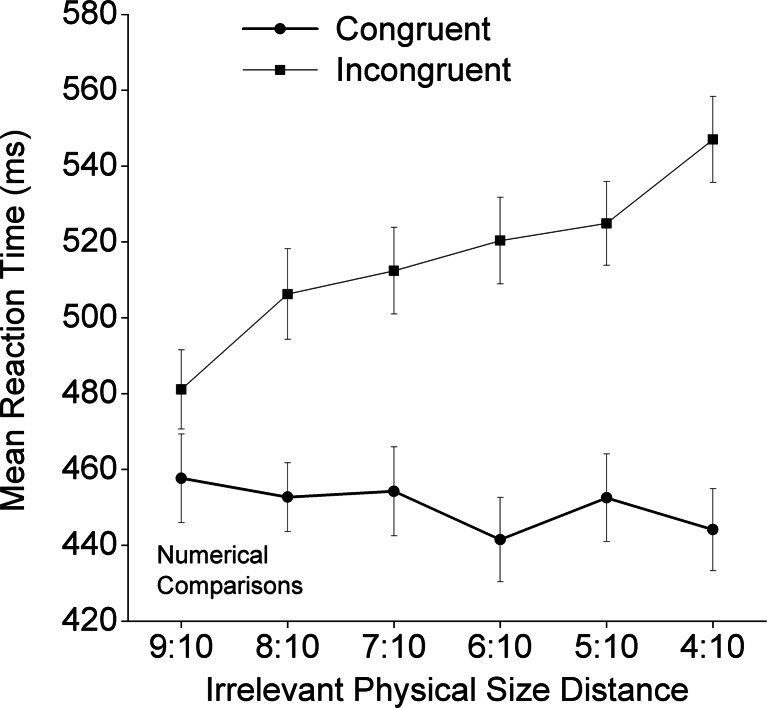
Mean reaction times and standard errors in the numerical task as a function of congruity and the task-irrelevant physical size distance

In the numerical task, the interaction among congruity, physical size distance, and numerical distance was significant [*F*(5, 175) = 6.16, adjusted *p* < .001]. The main effect of numerical distance was significant [*F*(1, 35) = 111.92, adjusted *p* < .001], as was its interaction with congruity [*F*(1, 35) = 30.26, adjusted *p* < .001; see Fig. 3]. Although the congruity effect was significant for both the close and far conditions, the effect appeared greater in the close than in the far condition [close, *F*(1, 35) = 210.28, adjusted *p* < .001; far, *F*(1, 35) = 125.34, adjusted *p* < .001]. Both physical size distance and congruity had significant main effects on the mean RTs [*F*(5, 175) = 19.23, adjusted *p* < .001, and *F*(1, 35) = 190.30, adjusted *p* < .001, respectively]. Their interaction was also significant [*F*(5, 175) = 38.11, adjusted *p* < .001; see Fig. 4]. To further investigate the significant interaction between congruity and physical size distance, we performed simple-effect analyses to examine the task-irrelevant physical size distance effects for the congruent and incongruent conditions separately. Under the congruent condition, we observed a significant and positive physical size distance effect [*F*(5, 31) = 5.82, adjusted *p* < .01]. Under the incongruent condition, we instead found a significant and negative physical size distance effect [*F*(5, 31) = 45.11, adjusted *p* < .001].

**Fig. 5:**
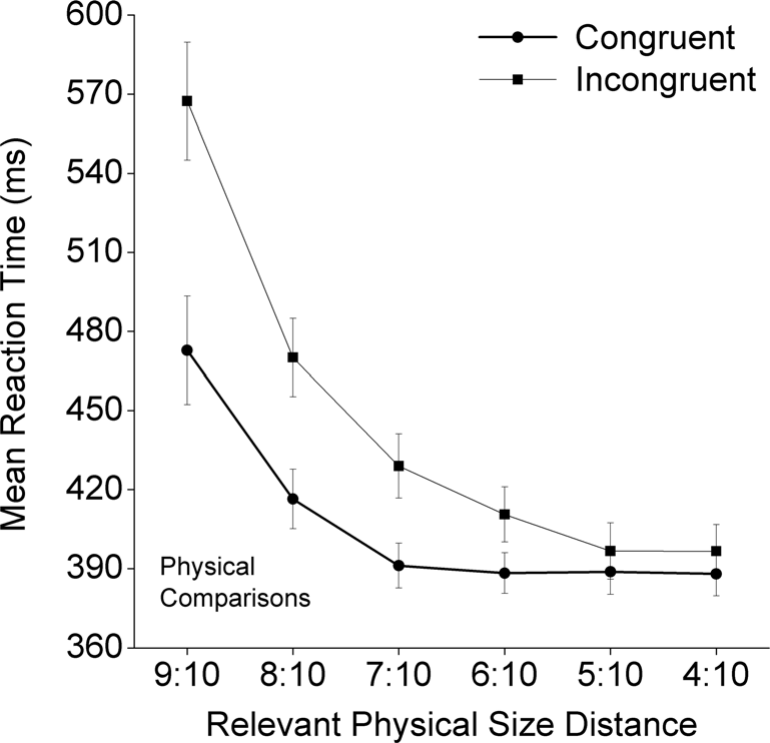
Mean reaction times and standard errors in the physical task as a function of congruity and the task-relevant physical size distance

**Fig. 6:**
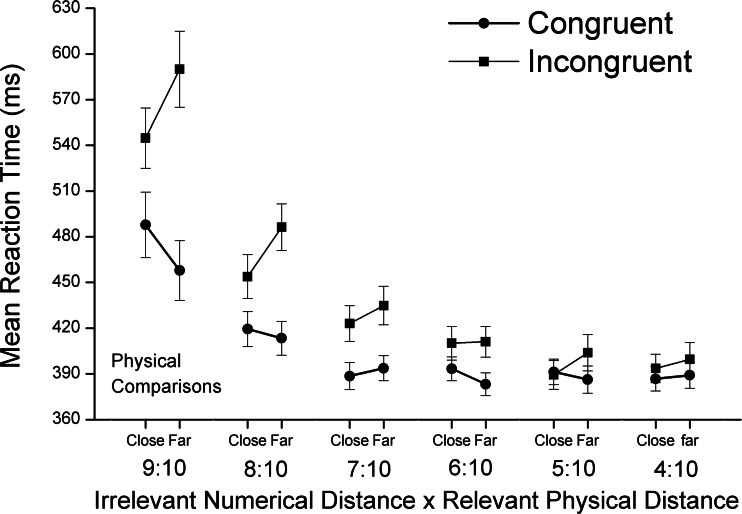
Task-irrelevant negative numerical distance effect (for the incongruent condition) and task-irrelevant positive numerical distance effect (for the congruent condition) for the physical comparison task as a function of physical size distance

In the physical task, the interaction among congruity, numerical distance, and physical size distance was significant [*F*(5, 175) = 11.98, adjusted *p* < .001]. The main effect of physical size distance was significant [*F*(5, 175) = 86.45, adjusted *p* < .001], as was its interaction with congruity [*F*(5, 175) = 37.50, adjusted *p* < .001]: The congruity effect was reduced with decreasing physical size ratio (see Fig. 5). Both numerical distance and congruity had significant main effects on the mean RTs [*F*(1, 35) = 5.55, adjusted *p* < .05; *F*(1, 35) = 66.61, adjusted *p* < .001, for numerical distance and congruity, respectively]. Their interaction was also significant [*F*(1, 35) = 32.99, adjusted *p* < .001]. Simple-effect analyses showed that, under the congruent condition, the positive numerical distance effect was significant [*F*(1, 31) = 10.77, adjusted *p* < .01], and under the incongruent condition, the negative numerical distance effect was also significant [*F*(1, 35) = 20.69, adjusted *p* < .001]. The Congruity × Numerical Distance × Physical Size Distance interaction was significant [*F*(5, 175) = 11.98, adjusted *p* < .001]. Following the procedure used by Pinhas, Tzelgov, and Ganor-Stern (2012), we conducted a trend analysis of the effect sizes. The results showed a significant decreasing linear trend of the interaction between congruity and task-irrelevant numerical distance [*F*(1, 35) = 7.78, *p* < .01; see Fig. 6]. Specifically, the interaction effects were significant for the close physical size distance levels [9:10, *F*(1, 35) = 31.22, adjusted *p* < .001; 8:10, *F*(1, 35) = 21.16, adjusted *p* < .001], but not for the other physical size distance levels (all adjusted *p*s > .005). To explore the interaction of congruity and numerical distance, we examined the task-irrelevant numerical distance effect on each physical size distance level for the congruent and incongruent conditions separately. The negative distance effect in the incongruent condition was statistically significant for the 9:10 trials [*F*(1, 35) = 14.81, adjusted *p* < .001] and the 8:10 trials [*F*(1, 35) = 16.69, adjusted *p* < .001], whereas the positive distance effect in the congruent condition was significant only for the 9:10 trials [*F*(1, 35) = 10.98, adjusted *p* < .005].

Although this was tangential to our study, we also examined whether the congruity effect was derived from interference or facilitation and whether the results varied by physical distance (Kallai & Tzelgov, 2012; see also MacLeod, 1991, for a review of the Stroop effect that discusses the difference between interference and facilitation). Following the procedure used by Kallai and Tzelgov (2012), we conducted repeated measures ANOVAs excluding numerical distance, for the numerical and physical tasks separately. We found the typical pattern of greater interference than facilitation (as was also found in Kallai & Tzelgov, 2012), which was modulated by the physical size distance. Specifically, the main effect of effect direction (facilitation vs. interference) was significant [numerical task, *F*(1, 35) = 31.32, *p* < .001; physical task, *F*(1, 35) = 22.46, *p* < .001], as was its interaction with physical size distance [numerical task, *F*(5, 175) = 19.23, *p* < .001; physical task, *F*(5, 175) = 5.82, *p* < .001].

The overall error rate was low (2.95 %). In the numerical task, errors increased from 0.47 % to 1.44 % to 8.38 % across the congruent, neutral, and incongruent conditions. In the physical task, the error rates were 0.79 %, 1.24 %, and 5.38 % under the congruent, neutral, and incongruent conditions, respectively. A correlation analysis was conducted between RTs and error rates and showed that there was little RT–accuracy trade off (*r* = .13).

## Discussion

We investigated the impacts of numerical distance (close vs. far), physical size distance (from 9:10 to 4:10), and congruity (congruent, neutral, and incongruent) on RTs in the number Stroop paradigm. The main results included: (a) For both numerical and physical tasks, main effects emerged of both congruity and task-relevant distance; (b) the congruity effect was reduced with increasing numerical distance (i.e., an overadditive interaction) for the numerical task, and with increasing physical size distance (from 9:10 to 4:10) for the physical task; and (c) a reliable positive task-irrelevant distance effect was apparent under the congruent condition, as well as a reliable negative task-irrelevant distance effect under the incongruent condition (i.e., the underadditive interaction; see Fig. 4 for the numerical task and Fig. 6 for the physical task, with the latter effect being mainly driven by the 9:10 and 8:10 physical size distance levels).

### Asymmetry versus symmetry

A main pattern of results from the classic color-naming Stroop studies is the asymmetry of faster word reading than color naming (MacLeod, 1991). According to the relative-speed-of-processing account, the faster task-irrelevant dimension should interfere more with the slower task-relevant dimension (MacLeod, 1991, p. 189). Therefore, the above asymmetry could explain the reliable interference when participants have to name the presentation color (i.e., the irrelevant word meaning interferes with naming the color), but less reliable interference when participants were asked to read the color words (i.e., the irrelevant color does not interfere with processing the word meaning). Researchers have mainly used three manipulations to reduce the asymmetry between word reading and color naming: practicing the color-naming response extensively (e.g., MacLeod & Dunbar, 1988), reducing the legibility of the word (e.g., Gumenik & Glass, 1970), and previewing the slower dimension (e.g., Glaser & Glaser, 1982; Glaser & Düngelhoff, 1984). However, the existing results showed that such manipulations have not always succeeded in classic color-word Stroop tasks.

Compared with color-word Stroop studies, the results from number Stroop studies are less asymmetrical. In other words, the congruity effects are significant and robust in both the numerical and physical tasks (Girelli et al., 2000; Henik & Tzelgov, 1982; Kaufmann et al., 2005; Rubinsten et al., 2002; Szűcs & Soltész, 2007; Szűcs, Soltész, Jármi & Csépe, 2007). Furthermore, the asymmetry between the task-relevant and task-irrelevant dimensions can be reduced by manipulating the physical size distance, as was done in the present study as well as in previous studies (Algom et al., 1996; Cohen Kadosh et al., 2008; Noël et al., 2005; Schwarz & Ischebeck, 2003).

We can draw three conclusions from the results of our study. First, the congruity effects found in both numerical and physical tasks implied that not only physical size, but also numerical value, is processed automatically. Second, with the relatively short mean RTs (~380 ms), the interference for physical comparisons from the task-irrelevant numerical-value dimension reached its limit, whereas the interference for numerical comparisons from the task-irrelevant physical size did not (see Fig. 2). Third, for the physical size distances of 8:10 and 9:10 (see Figs. 1 and 2), we observed, respectively, a symmetric pattern (i.e., in both physical and numerical tasks, influence from the task-irrelevant dimension on the task-relevant dimension were similar) and a reversed, asymmetric pattern (i.e., the physical-size dimension interfered with, rather than being interfered with by the numerical-value dimension). In the following section, we discuss the necessity of reducing the asymmetry between the task-relevant and task-irrelevant dimensions, particularly for detecting the task-irrelevant positive and negative effects of numerical distance on the potential underadditive interaction in physical comparisons.

### Dissociation of the congruity and distance effects

Many number Stroop studies have demonstrated that the congruity effect was found for both numerical and physical tasks (e.g., Henik & Tzelgov, 1982; Rubinsten et al., 2002; Tzelgov et al., 1992), but some studies have shown that the numerical distance effect was found only for numerical judgment, not for physical size judgment in adults (Girelli et al., 2000; Rubinsten et al., 2002; Tzelgov et al., 1992). Furthermore, Rubinsten et al. (2002) found an asymmetrical pattern—that is, a distance effect without a size congruity effect—in children at the beginning of the first grade. Rubinsten et al. suggested that such a dissociation of the congruity and numerical distance effects was evidence for their two-representation model.

Our study was designed to investigate whether the apparent dissociation was due to the asymmetrical designs of and an analytical issue in the previous studies. With improvements in the study design and analytical approach, we could show that there was no dissociation between congruity and numerical distance in adults. Therefore, it is possible that Rubinsten et al.’s (2002) finding (i.e., no numerical distance effect for the physical task) was due to their aggregation of the data from all three Stroop conditions: the congruent, neutral, and incongruent conditions in physical comparisons. Their way of aggregating the data across conditions had two potential problems. First, it included the neutral condition in the examination of the numerical distance effect, even though this condition did not involve any numerical distance. Second, the combination of the incongruent and congruent conditions might also have obscured potential interaction effects (the underadditive interaction), which was indeed what we found (see Figs. 4 and 6). Consistent with our argument, previous studies (e.g., Cohen Kadosh et al., 2008; Henik & Tzelgov, 1982; Ito & Hatta, 2003) have shown, but have not paid much attention to, very similar interactive effects between numerical distance and congruity conditions.

**Fig. 7:**
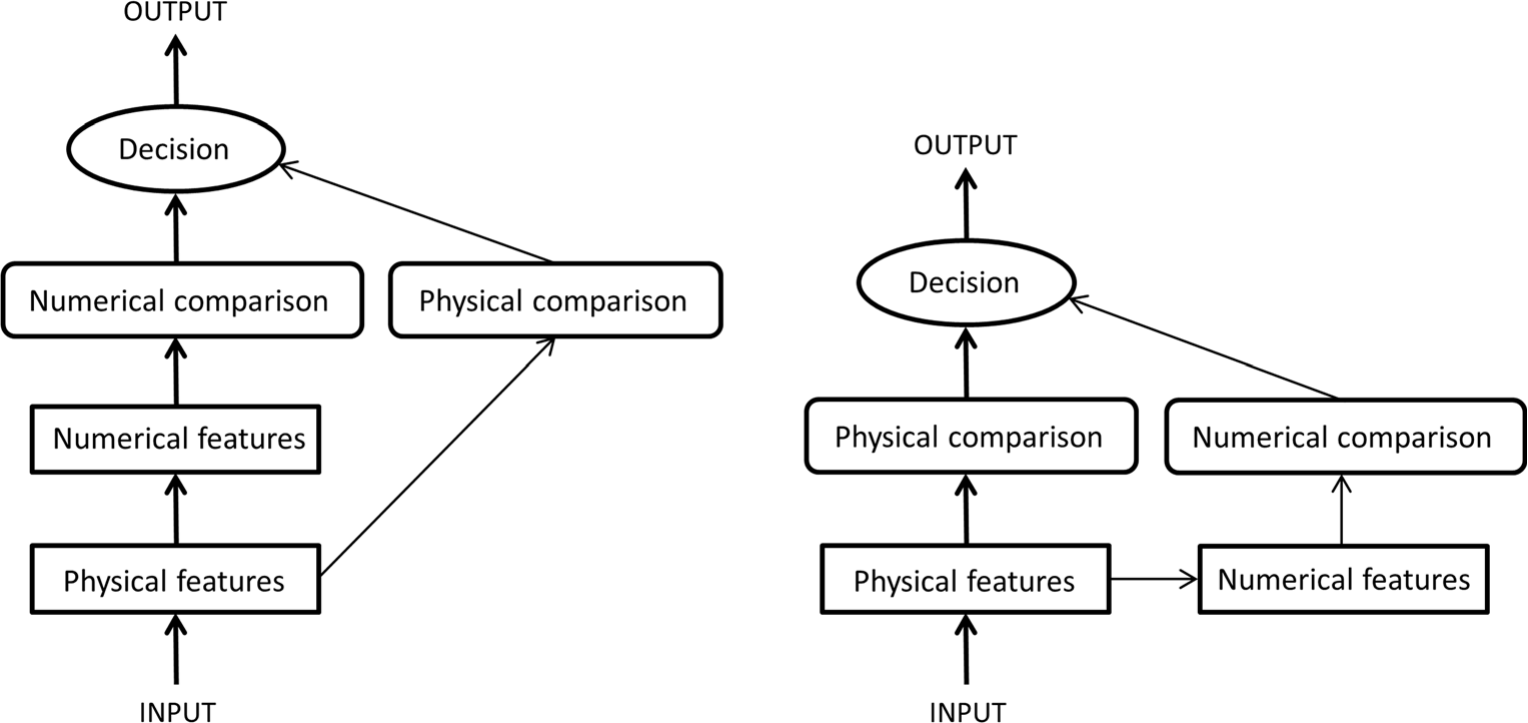
Architectures for models of number-size Stroop interference for numerical comparisons (left panel) and physical comparisons (right panel). The processing of the task-relevant stimulus dimension is represented by the bold black arrows and boxes, whereas the processing of the task-irrelevant dimension is represented by thin black arrows and boxes

When we directly tested whether the task-irrelevant numerical distance effect occurred under either the congruent or the incongruent condition, the observed pattern (see Fig. 6) provided a possible explanation for why most of the previous number Stroop studies, particularly those using the selection version, did not detect the task-irrelevant numerical distance effect in the physical task. That is, these previous studies were based on an asymmetrical design, with only one physical size distance level, and did not attempt to reduce or reverse the asymmetry of the task-relevant and task-irrelevant dimensions (faster physical size processing vs. slower numerical value processing). In the present study, we showed that, with a symmetrical design and a “reversed” asymmetrical design (i.e., by creating more physical size distance levels, including the “close” distances of 8:10 and 9:10), we could detect the effects of the task-irrelevant dimension on the potential underadditive interaction (Fig. 7).

In summary, our results suggest that the lack of attention to the potential interactions and the use of an asymmetrical design might have contributed to some earlier findings of the dissociation between the congruity and numerical-distance effects. Because such findings have provided the main evidence for Rubinsten et al.’s (2002) two-representation model of numerical magnitude processing, our study calls for a reevaluation of their model and adds empirical support to several researchers who have aired doubts concerning the validity of the two-representation model in explaining the data on the development of children’s magnitude representation (for a detailed discussion, see Noël et al., 2005, p. 189).

## Conclusion

To summarize, in the present study we attempted to reevaluate the two-representation model of numerical magnitude processing. Our results indicate that the seemingly contradictory findings in previous studies can be traced to methodological deficiencies and a biased asymmetrical design exemplified in Rubinsten et al.’s (2002) study. We have provided evidence in favor of Dehaene and Akhavein’s (1995) original conclusions about the automaticity of number magnitude processing under the theoretical framework of Schwarz and Ischebeck’s (2003) coalescence model.
